# Gene Expression and Levels of TGF-B in PBMC Is Associated with Severity of Symptoms in Chronic Heart Failure

**Published:** 2020

**Authors:** Samaneh Saadati, Vajiheh Eskandari, Farzaneh Rahmani, Mohammad Jafar Mahmoudi, Zahra Rahnemoon, Zahra Rahmati, Fatemeh Gorzin, Mona Hedayat, Ali Akbar Amirzargar, Nima Rezaei

**Affiliations:** 1. Department of Immunology, Faculty of Medicine, Tehran University of Medical Sciences, Tehran, Iran; 2. Cellular and Molecular Research Center, Faculty of Medicine, Guilan University of Medical Sciences, Rasht, Iran; 3. Research Center for Immunodeficiencies, Pediatrics Center of Excellence, Children’s Medical Center, Tehran University of Medical Sciences, Tehran, Iran; 4. NeuroImaging Network (NIN), Universal Scientific Education and Research Network (USERN), Tehran, Iran; 5. Department of Cardiology, Amir Alam Hospital, Tehran University of Medical Sciences, Tehran, Iran; 6. Cardiac Heart Center, Faculty of Medicine, Tehran University of Medical Sciences, Tehran, Iran; 7. Division of Immunology, Boston Children’s Hospital, Harvard Medical School, Boston, MA, USA; 8. Network of Immunity in Infection, Malignancy and Autoimmunity (NIIMA), Universal Scientific Education and Research Network (USERN), Boston, MA, USA

**Keywords:** Cell culture techniques, Chronic heart failure, T-lymphocytes, Transforming growth factor beta1

## Abstract

**Background:**

TGF-β1 is known to promote cardiac remodeling and fibrosis during Congestive Heart Failure (CHF). In this study, an attempt was made to investigate expression of Transforming Growth Factor beta1 (TGF-β1) and relative expansion or contraction of regulatory T-cell (Tregs) population in peripheral blood of patients with Chronic Heart Failure (CHF).

**Methods:**

Real-time PCR assay was used to investigate expression and post-stimulation levels of TGF-β1 in cell culture supernatant of Peripheral Blood Mononuclear Cells (PBMC) of 42 patients with CHF and 42 controls. Flow cytometry was used to identify relative counts of CD4^+^CD25^+^FoxP3^+^ Tregs.

**Results:**

PBMCs in patients with CHF expressed higher levels of TGF-β1 compared to controls. Post-stimulation levels of TGF-β1 expression were significantly higher in New York Heart Association (NYHA) functional class IV patients compared to stage I patients. Tregs were significantly expanded in PBMC in CHF, while the CD4^+^ helper T-cells were unchanged. Treg expansion was more significant in NYHA functional class I patients compared to class IV patients.

**Conclusion:**

Expansion of Treg population in CHF provides an extrinsic source for TGF-β1 production to induce reactive fibrosis and cardiac remodeling. Relative decrease in Treg population at advanced stages of CHF is indicative of a loss of regulatory characteristics in these cells and unopposed proinflammatory milieu.

## Introduction

Glazer *et al* first demonstrated an increase in Transforming Growth Factor beta (TGF-β1) expression as an early event in Congestive Heart Failure (CHF) 1. Elevated serum levels of TGF-β1 are also associated with increased risk of heart failure, as demonstrated through a cohort on “Cardiovascular Health Study” 1. Members of the TGF family including TGF-β1, soluble TGF-β and Smad2 2, and Latent TGF-β Binding Protein (LTBP) 3 are potential new biomarkers in heart failure. Sources for these peripheral cytokines are cardiomyocytes and cardiac fibroblasts themselves as well as monocyte and circulating lymphocytes 4. Also, levels of plasma cytokine expression in Peripheral Blood Mononuclear cells (PBMC) are reflected in cardiomyocyte and cardiac fibroblasts 5. Gene expression of proinflammatory cytokines has been previously investigated in PBMC of patients with CHF 6,7. Therefore, the same approach investigating expansion/contraction of regulatory T-cells (Treg) subtypes and TGF-β expression by these cells was followed in this study.

## Materials and Methods

A case-control study was designed enrolling 42 patients (24 males; age=55.3±5.76 years) with CHF from referrals to the Tehran Heart Center, and another 42 matched, healthy individuals (26 males; age=54.35± 0.74) as the control group. The diagnosis of CHF in case group was made on clinical grounds as well as the patient’s most recent echocardiography in which the Left Ventricular Ejection Fraction (LVEF) had been measured less than 45%. Mean LVEF of the patients was 24.6±1.13% with a mean systolic/diastolic blood pressure of 143±30/103±20 *mmHg*. Patients were excluded if they were in decompensated heart failure phase at the time or within the last 1 month of sampling, or if they had experienced acute coronary syndrome within the last 6 months.

5 *ml* whole blood was obtained to isolate PBMC through Ficoll–Paque density gradient centrifugation. Total cellular RNA was isolated from 5–10 ×10^6^ cells from the PBMC solution by 12000 RPM centrifugation, using RNX solution (SinaClon, Tehran, Iran). Next, 1 *μg* of total RNA from each sample was reverse transcribed to complementary DNA (cDNA) using First Strand High Capacity cDNA Reverse Transcription Kit (CinnaGen, Tehran, Iran). TGF-β mRNA expression levels were analyzed using a quantitative real-time Reverse Transcriptase Polymerase Chain Re-action (RT-PCR) method by TaqMan Universal PCR Master Mix (ABI, London, UK). Differences in gene expression were normalized against β Actin as a housekeeping gene, *via* ABI Prism7900 Sequence Detection System (Applied Biosystems, USA) 8.

Next, 1×10^6^ cells from each tube of cultured PBMC were stained with anti-cell-surface antibodies, anti-CD4-FITC and anti-CD25-PE antibodies (Biolegend, Germany, 317416 and 302606). The resulting cellular cocktail was used to obtain flow cytometry data on a FACS Calibur (Pharmacia, Sweden) using the Flowjo 7.6 software. Statistical analyses were performed using SPSS software version 23.

## Results

**Figure 1.**
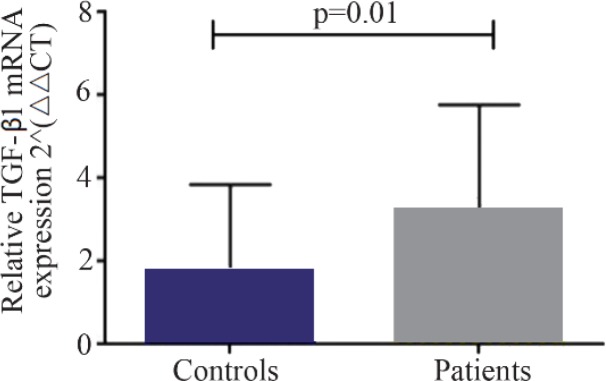
Baseline transforming growth factor beta 1 (TGF-β1) gene expression in peripheral blood mononuclear cells of patients with chronic heart failure and healthy controls, 2^−ΔΔCT^: comparative delta delta cycle threshold method.

**Figure 2.**
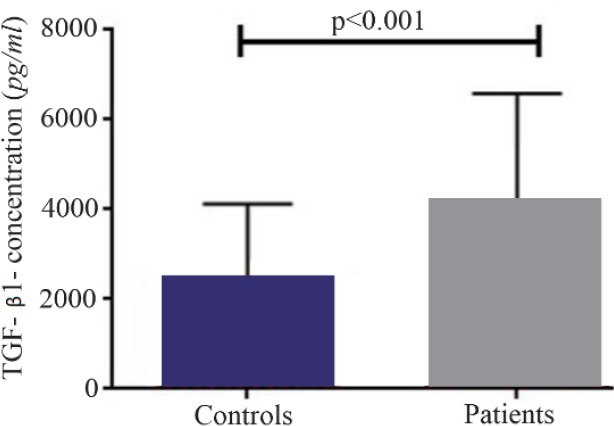
Baseline transforming growth factor beta 1 (TGF-β1) level in supernatant of peripheral blood mononuclear cells cultures of patients with chronic heart failure and healthy controls, following incubation with PHA 1.5%.

**Figure 3.**
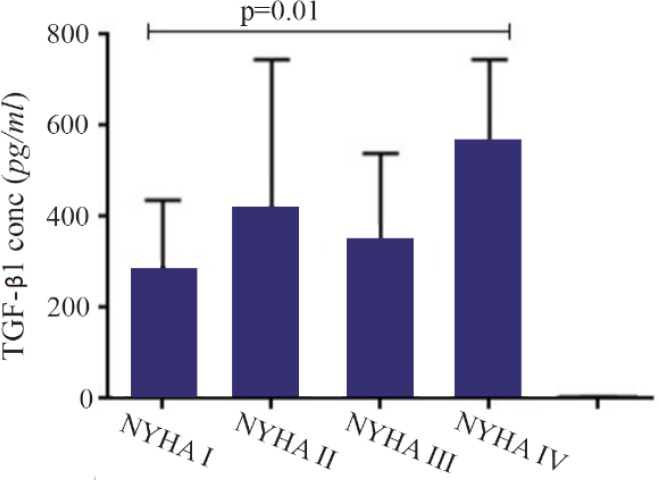
Transforming growth factor beta 1 (TGF-β1) gene expression in peripheral blood mononuclear cells of patients with chronic heart failure with different functional classes based on New York Heart Association (NYHA) classification, following incubation with PHA 1.5%.

**Figure 4.**
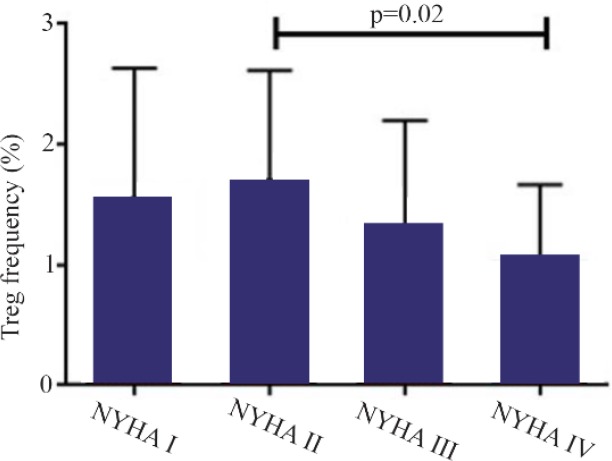
Absolute count of CD4^+^CD25^+^FoxP3^+^ regulatory T-cells of patients with chronic heart failure with different functional classes based on New York Heart Association (NYHA) classification, following incubation with PHA 1.5%, CD=cluster of differentiation.

A significant increase in mRNA expression of TGF-β1 was found in patients with CHF (3.1±2.66) compared to controls (1.75±2.11) (p=0.01) (Figure 1). Higher TGF-β1 expression in patients with CHF was not dependent on the etiology of ischemia, nor was it different between patients in different NYHA classes. PBMCs of patients with CHF and upregulated TGF-β1 expression following stimulation with PHA were significantly more than the ones in healthy controls (p< 0.001) (Figure 2). Stimulated TGF-β1 expression was significantly higher in patients in NYHA functional class IV compared to those in class I (p=0.01) (Figure 3). There was no difference in TGF-β1 expression between patients with ischemic versus non-ischemic origin of CHF. Finally, the absolute count of CD4^+^ CD25^+^FoxP3^+^ T-cells (Regulatory T-cells) was increased in patients with CHF compared to controls (p= 0.01). The percentage of CD4^+^ helper T-cells was unchanged in patients with CHF (41.04±2.35 in control versus 38.5±2.68 in CHF; p=0.2), yet the relative count of regulatory over helper T-cells was significantly increased in patients with CHF compared to healthy controls (Treg/CD4^+^ ratio=2.06±1.26 and 1.34±0.85 in CHF versus control, p=0.02). Interestingly, patients with CHF in NYHA class IV had a significantly lower count of CD4^+^CD25^+^FoxP3^+^ T-cells compared to those in class I (p=0.02) (Figure 4).

## Discussion

Our results confirm previous findings on peripheral TGF-β1 upregulation in chronic heart failure and the possible benefits in suppressing TGF-β expression to prevent cardiac muscle fibrosis. TGF-β expression did not show any correlation with patient’s functional class at baseline level, but did so following stimulation of cultured PBMC with PHA. Lymphocytes of CHF patients expressed lower TGF-β during early functional stages of disease, compatible with relative expansion of CD4^+^CD25^+^FoxP3^+^ regulatory T-cells over CD4^+^ helper T-cells in CHF. Paradoxically, this expansion was less prominent in patients with NYHA functional class IV compared to those in class I, and was most prominent in NYHA class II. This could be justified in a two phase model of cytokine expression in CHF. In patients with well-compensated CHF, peripheral cytokines are largely from cardiac origin which later stimulate polarization of T lymphocyte into regulatory phenotype. Expansion of Treg population and relative contraction of helper T-cells are then responsible for excessive amounts of TGF-β produced in later stages of CHF and progressive fibrosis. In line with this, no difference was previously shown in expression of FoxP3, interleukin 10 and Treg transcription factors in early CHF 9.

An expansion in baseline expression of TNF-α and IL-6 as two major proinflammatory cytokines in PBMC of CHF has been previously identified 6. The potential of PBMC to express IL-6 and TNF-α increased along with disease progression 6, suggesting a compensatory role for these cytokines in response to progressive fibrosis and accumulation of profibrotic cytokines, TGF-β, angiotensin II, *etc*. According to Okamoto *et al*, compensatory increase in IL-6 expression is in favor of naïve T-cells to maturate into Th17 subtype rather than Treg phenotype, counteracting profibrotic function in advanced stages of CHF 10. Okamoto *et al* also suggested a prognostic value for Treg, predicting a more rapid decline in ventricular ejection fraction in patients with CHF 10.

## Conclusion

Our results are in line with previous findings that early inhibition of TGF-β is in favor of production of proinflammatory cytokines and neutrophil infiltration, while late TGF-β blockade has anti-fibrotic, anti-remodeling effects.
